# An Endogenous Retroviral LTR-Derived Long Noncoding RNA lnc-LTR5B Interacts With BiP to Modulate ALV-J Replication in Chicken Cells

**DOI:** 10.3389/fmicb.2021.788317

**Published:** 2021-11-29

**Authors:** Shihao Chen, Ruihan Zhao, Ting Wu, Dedong Wang, Biao Wang, Shiyu Pan, Xuming Hu, Zhiming Pan, Hengmi Cui

**Affiliations:** ^1^Institute of Epigenetics and Epigenomics and College of Animal Science and Technology, Yangzhou University, Yangzhou, China; ^2^Joint International Research Laboratory of Agricultural & Agri-Product Safety, The Ministry of Education of China, Yangzhou University, Yangzhou, China; ^3^Jiangsu Key Laboratory of Zoonosis, Yangzhou University, Yangzhou, China; ^4^College of Veterinary Medicine, Yangzhou University, Yangzhou, China; ^5^Institute of Comparative Medicine, Yangzhou University, Yangzhou, China

**Keywords:** ALV-J, lncRNA, lnc-LTR5B, BiP, interaction

## Abstract

Infection with the avian leukosis virus subgroup J (ALV-J) impairs host genes and facilitates the establishment of chronic infection and the viral life cycle. However, the involvement of long noncoding RNAs (lncRNAs) in ALV-J infection remains largely unknown. In this study, we identified a novel chicken lncRNA derived from LTR5B of the ERV-L family (namely lnc-LTR5B), which is significantly downregulated in ALV-J infected cells. lnc-LTR5B was localized in the cytoplasm and was relatively high expressed in the chicken lung and liver. Notably, the replication of ALV-J was inhibited by the overexpression of lnc-LTR5B but enhanced when lnc-LTR5B expression was knocked down. We further confirmed that lnc-LTR5B could bind to the binding immunoglobulin protein (BiP), a master regulator of endoplasmic reticulum (ER) function. Mechanistically, lnc-LTR5B serves as a competing endogenous RNA for BiP, restricting its physical availability. Upon ALV-J infection, the reduction of lnc-LTR5B released BiP, which facilitated its translocation to the cell surface. This is crucial for ALV-J entry as well as pro-survival signaling. In conclusion, we identified an endogenous retroviral LTR-activated lnc-LTR5B that is involved in regulating the cell surface translocation of BiP, and such regulatory machinery can be exploited by ALV-J to complete its life cycle and propagate.

## Introduction

Avian leukosis virus (ALV), which belongs to the family *Retrovirus*, is an enveloped RNA virus that causes host immunosuppression and various tumors in chicken. ALVs have been classified into seven subgroups, including ALV-A, B, C, D, E, J, and K (Chai and Bates, 2006; Li et al., 2016). ALV-J displays higher transmissibility and pathogenicity than other subgroups, which has been considered as one of the leading causes of morbidity and mortality in some chicken flocks in China (Li et al., 2019a,b); thus, it presents a great threat to the poultry industry and leads to high economic loss. The lack of understanding of the viral life cycles has hampered the development of specific antiviral drugs or effective vaccines against ALV-J. Therefore, it is important to discover the underlying molecular mechanisms of ALV-J replication and disease progression. Successful ALV-J infection requires host factors at different stages of the viral life cycle. For example, ALV-J infection begins with the viral envelope (Env) protein binding to its specific receptor NHE1 at the surface of host cells, followed by virion internalization (Chai and Bates, 2006; Guan et al., 2018). In addition, ALV-J often has multiple mechanisms to interfere with the host innate immune response. For example, MiR-34b-5p, which is induced by ALV-J infection, targets melanoma differentiation-associated gene 5 (MDA5), which is a major cellular sensor for triggering type I interferon (Li et al., 2017). ALV-J infection blocks the activation of the type I interferon signaling by targeting the transcriptional regulator NF-kB (Lin et al., 2018). Therefore, screening more host factors targeted by ALV-J is beneficial to understand its pathogenic mechanisms and develop effective vaccines.

Long noncoding RNAs (lncRNAs) are transcripts that are longer than 200 nucleotides in length and possess no protein-coding properties. Although they do not code for proteins, lncRNAs are considered to play pivotal roles in diverse biological processes (Carpenter et al., 2013; Durruthy-Durruthy et al., 2016; Olivero et al., 2020). Recently, many lncRNAs have been shown to be associated with host defense responses against viral infections. For example, lncRNA Malat1 suppresses antiviral innate responses by interacting with TDP43 and affecting its cleavage, which is mediated by activated caspase-3 (Liu et al., 2020). The lncRNA NRAV promotes respiratory syncytial virus (RSV) replication by sponging miR-509-3p to release Rab5c and promote RSV vesicle transportation (Li et al., 2020). Our previous research suggested that a chicken lncRNA named lnc-ALVE1-AS1 inhibits ALV-J replication by simulating TLR3 signaling and promoting the transcription of interferon-stimulated genes (Chen et al., 2019). We and others have indicated that numerous lncRNAs were significantly differentially expressed in chicken cells during ALV-J infection (Qiu et al., 2017; Hu et al., 2018; Dai et al., 2019). Among these lncRNAs, some were predicted to be involved in antiviral signaling, but very few have been functionally characterized.

In this study, we identified a chicken endogenous retrovirus LTR-derived lncRNA, lnc-LTR5B, whose expression is downregulated during ALV-J infection. We demonstrated that the overexpressed lnc-LTR5B exhibited an inhibitory effect on ALV-J replication *in vitro*. Our findings suggested that lnc-LTR5B binds to BiP in the cytoplasm, and the reduction of lnc-LTR5B in ALV-J infection is part of establishing the virus cycle by increasing BiP translocation to the plasma membrane and ALV-J entry. Our study provides a plausible, virus-modulating, lncRNA-based disease mechanism that opens avenues for developing new antiviral strategies.

## Materials and Methods

### Cells and Virus

Primary chicken embryo fibroblast cells (CEFs) were prepared from 10-day-old specific pathogen-free chicken embryos, following our approved protocol. The CEFs and DF-1 cells (ATCC and CRL-12203) were maintained in Dulbecco’s modified Eagle’s medium (GIBCO) supplemented with 10% fetal bovine serum (FBS; GIBCO), penicillin (100U/ml), and streptomycin (100μg/ml) at 37°C in a humidified 5% CO_2_ incubator.

The ALV-J (JS09GY3 strain) and the monoclonal antibody JE9 against ALV-J Env (gp85) were kindly provided by Prof. Aijian Qin (Yangzhou University, China). The 50% tissue culture infective doses (TCID_50_) of the ALV-J virus were measured in DF-1 cells using the Reed and Muench method. ALV-J RNA was quantified by qPCR using Env gene-specific primers, and the results were normalized to the expression levels of GAPDH.

### RNA Extraction and Quantitative Real-Time PCR

Total RNA was extracted from cells or eight kinds of chicken normal tissues using TRIzol reagent (Takara). cDNA was synthesized using a Vazyme cDNA Synthesis Kit with gDNA Eraser Kit (Vazyme, Nanjing, China) according to the manufacturer’s protocol. qPCRs were performed with the ChamQ SYBR qPCR Master Mix Kit (Vazyme) on a BioRad CFX Connect Real-time PCR detection system (Bio-Rad). The relative expression levels were normalized with the GAPDH gene by the comparative Ct (2^−∆∆Ct^) method. The primers sequences for qRT-PCR are listed in Supplementary Table S1.

### Rapid Amplification of cDNA Ends (5' and 3' Race)

5' RACE and 3' RACE were performed with SMARTer RACE5'/3' Kit (Takara) according to the manufacturer’s protocol. Briefly, total RNA from CEF cells was isolated by using TRIzol reagent (Takara) and was reverse-transcribed into the first-strand cDNA using 5' or 3' end primers. Then, the cDNA was used as template for PCR amplification to generate 5' or 3'-end of the clone. 5'- and 3'-specific primer are listed in Supplementary Table S1.

### Plasmids Construction

To generate pcDNA3.1-lnc-LTR5B, the full-length lnc-LTR5B was PCR-amplified and cloned into a pcDNA3.1 plasmid with the MultiF Seamless Assembly Mix (ABclonal) according to the manufacturer’s instructions. The different DNA fragments of lnc-LTR5B promoter were PCR-amplified using the following pairs of primers: FL-F and FL-F for FL region; T1-F and FL-F for T1 region; T2-F and FL-F for T2 region; and FL-F and T3-R for T3 region. Then, these DNA fragments were cloned into the pGL3-basic vector (Promega) to generate the reporter construct. The oligo sequences for the plasmid construct are listed in Supplementary Table S1.

### Luciferase Reporter Assay

2×10^5^ 293T cells were seeded in 24-well plates and cotransfected with a control plasmid pRL-TK (Promega) and indicated plasmids for luciferase activity. After 36h of incubation, the cells were harvested and luciferase activities were measured using the Dual Luciferase Reporter Assay Kit (Vazyme), according to the manufacturer’s instructions. Relative luciferase signals (firefly/Renilla) were normalized against that of the pGL3-basic empty vector.

### ASO Transfection

The specific targeting lnc-LTR5B (ASO-1 and ASO-2) or non-targeting control ASO (ASO-NC) were designed and synthesized by RiboBio Company. ASO transfection was performed in 12-well plates with three technical replicates for each sample. Briefly, DF-1 cells were transfected with a final concentration of 50nM of each ASO until cell density reached ~50% confluence by riboFECT^™^ CP reagent (RiboBio) according to the manufacturer’s instruction. The ASO targeting sites are listed in Supplementary Table S1.

### Western Blot Analysis

Cells were lysed in a lysis buffer (Cell Signaling Technology) supplemented with the complete protease inhibitor cocktail (Roche) and incubated on ice for 15min. Protein concentrations were measured by the BCA Protein Assay kit (Beyotime). A total of 25 to 50μg of protein were separated by SDS-PAGE gel and transferred to a nitrocellulose membrane (Cytiva). The membranes were incubated at 4°C overnight with the appropriate primary antibodies: anti-BiP (HuaBio), anti-Bcl2 (Proteintech), anti-cleaved-caspase-3 (Proteintech), anti-GAPDH (Abcam), and anti-β-actin (Abcam). The images were captured with a FlourChem Q imaging system (ProteinSimple). The relative intensities of the different protein bands were analyzed using Image J program.

### Cell Fractionation

The membrane and cytoplasmic cell fractions were extracted using Membrane and Cytosol Protein Extraction Kit (Beyotime), according to the manufacturer’s protocol. Briefly, the treated cells were harvested and washed twice with PBS and then incubated with ice-cold Extraction Regent A containing protease inhibitor mixture on ice for 15min with gentle agitation. The supernatant (cytoplasmic fraction) was obtained by centrifugation at 700g at 4°C for 10min. And the sediment was resuspended with 200μl of ice-cold Extraction Regent B containing the protease inhibitor mixture. Finally, the supernatant was used as the membrane fraction to detect BiP protein expression.

### Viral Entry Assay

The viral entry assay was performed as described in a previous publication (Guan et al., 2018) with modifications. The cells were transfected with pcDNA3.1 or pcDNA3.1-lnc-LTR5B as described. After 24h post-transfection, the cells were washed twice with PBS, followed by incubation with ALV-J (MOI=5) at 37°C. After 2-h incubation, the cells were washed three times with pre-warmed PBS and then cultured in fresh DMEM supplemented with 2% FBS at 37°C with 5% CO2. The virus entry levels were measured at 12h post-infection by qRT-PCR for detecting ALV-J RNA.

## RNA Fish

Subcellular localization of lnc-LTR5B was assessed in DF-1 cells using Fluorescent *In Situ* Hybridization Kit (RiboBio) as described previously (Chen et al., 2019). The lnc-LTR5B probes labeled with Cy3 fluorescent dye were purchased from RiboBio Company. Briefly, DF-1 cells were fixed with 4% paraformaldehyde in PBS for 10min and permeabilized with 0.5% Triton X-100 for 10min. After blocking with pre-hybridization buffer/blocking solution, the cells were incubated with the lnc-LTR5B probes at 37°C overnight. The next day, cells were washed with appropriate saline sodium citrate buffer. The slides were then incubated with anti-BiP antibody at 4°C overnight. After washing three times with chilled PBS, the slides were incubated with Alexa Fluor 488-conjugated secondary antibody. Nuclei were then stained using DAPI (RiboBio) for 10min and finally analyzed by confocal microscopy (Leica SP8).

### RNA Pull Down and Mass Spectrometry

The DNA fragments of full-length lnc-LTR5B and its antisense were amplified with primers containing a T7 promoter sequences (listed in Supplementary Table S1). The PCR products were transcribed to RNA *in vitro* using the TranscriptAid T7 High Yield Transcription Kit (Thermo Fisher Scientific). Then, the biotinylated RNAs were produced using the Pierce RNA 3′ Desthiobiotinylation Kit (Thermo Scientific) and purified with phenol/chloroform/isoamyl alcohol, followed by ethanol precipitation. Next, 50pmol of biotin-labeled RNA was mixed with Pierce nucleic-acid compatible streptavidin magnetic beads in Protein-RNA Binding Buffer (Thermo Scientific) followed by incubation with DF-1 cell lysates for 1h with gentle agitation. After washing, the RNA-protein mixtures were extracted and further subjected to 12% SDS-PAGE for silver staining or western blotting. The selected bands were cut and subjected to mass spectrometry analyses.

### RNA Immunoprecipitation Assay

For RIP assay, 2×10^7^ DF-1 cells were washed twice with PBS and lysed in RIP lysis buffer (25mM Tris–HCl pH 7.4, 150mM KCl, 5mM EDTA, 0.5mM DTT, and 0.5% NP-40) supplemented with protease inhibitor cocktail and an RNase inhibitor, for 30min on ice. The lysates were then incubated with prewashed Protein A/G magnetic beads and coated with 5μg BiP (HuaBio) or normal Rabbit IgG (Abcam), with overnight shaking at 4°C. The next day, the beads were washed three times with ice-cold NT2 buffer (50mM Tris–HCl (pH 7.4), 150mM NaCl, 1mM MgCl2, and 0.05% NP-40) with protease inhibitor cocktail and an RNase inhibitor, to obtain the RNA-Protein complex. RNA was then isolated using TRIzol and detected by qRT-PCR.

### Immunofluorescence and Confocal Microscopy

DF-1 cells were grown on glass coverslips; after 24h, cells were washed with PBS three times. To visualize intracellular proteins, cells were fixed with 4% paraformaldehyde in PBS for 10min at room temperature (RT) and permeabilized with 0.25% Triton X-100 in PBS for 10min on ice. After blocking with 5% BSA and 2% donkey serum in PBS for 30min, the slides were then subjected to incubation with primary antibodies. To visualize cell surface proteins, cells were immediately fixed in 4% paraformaldehyde in PBS (4°C, 15min), followed by blocking with 5% BSA and 2% donkey serum in PBS (4°C, 30min). All primary antibodies diluted in PBS were incubated overnight at 4°C. The primary antibodies used were as follows: mouse anti-Env (1:300) and rabbit anti-BiP (1:200, HuaBio). The secondary antibodies used in this study were Alexa Fluor 488 donkey anti-rabbit antibody (1:1000; Invitrogen) and Alexa Fluor 594 donkey anti-mouse antibody (1,1,000; Invitrogen). The slides were washed three times with PBS, and nuclei were stained using DAPI (Sigma-Aldrich). Imaging was obtained using a Leica SP8 Confocal system with 63x oil objective and analyzed by the LAS X software version 3.0.2.16120.

### Statistical Analysis

The data are represented as mean±standard deviation (SD) of three independent experiments for each group and statistically analyzed by GraphPad Prism version 7.0 (GraphPad software). Statistical significance was assessed by Student’s t test or one-way ANOVA as shown in each figure legend. Differences with *p* values less than 0.05 were considered as statistically significant.

## Results

### Identification of a Host lncRNA, lnc-LTR5B, Which Is Downregulated in ALV-J-Infected Cells

To identify the host lncRNAs involved in ALV-J replication, we used RNA-seq to perform transcriptional analysis of CEF that were infected with ALV-J (JS09GY3 strain) or were mock-infected. The analysis data were presented in our previous work (Hu et al., 2018). Based on the idea that host lncRNAs targeted by viruses may participate in cellular antiviral responses, we focused on these downregulated lncRNAs during ALV-J infection. One of these lncRNAs, lnc-LTR5B (NCBI accession number: MG066622.1), was robustly downregulated in CEFs following ALV-J infection. RepeatMasker analysis showed that the MG066622.1 transcript has higher similarity with LTR5B elements, suggesting that lnc-LTR5B is an ERV-associated lncRNA. Considering that LTR contains a cis-regulatory region, the potential function of lnc-LTR5B in ALV-J infection requires urgent investigation.

**Figure 1:**
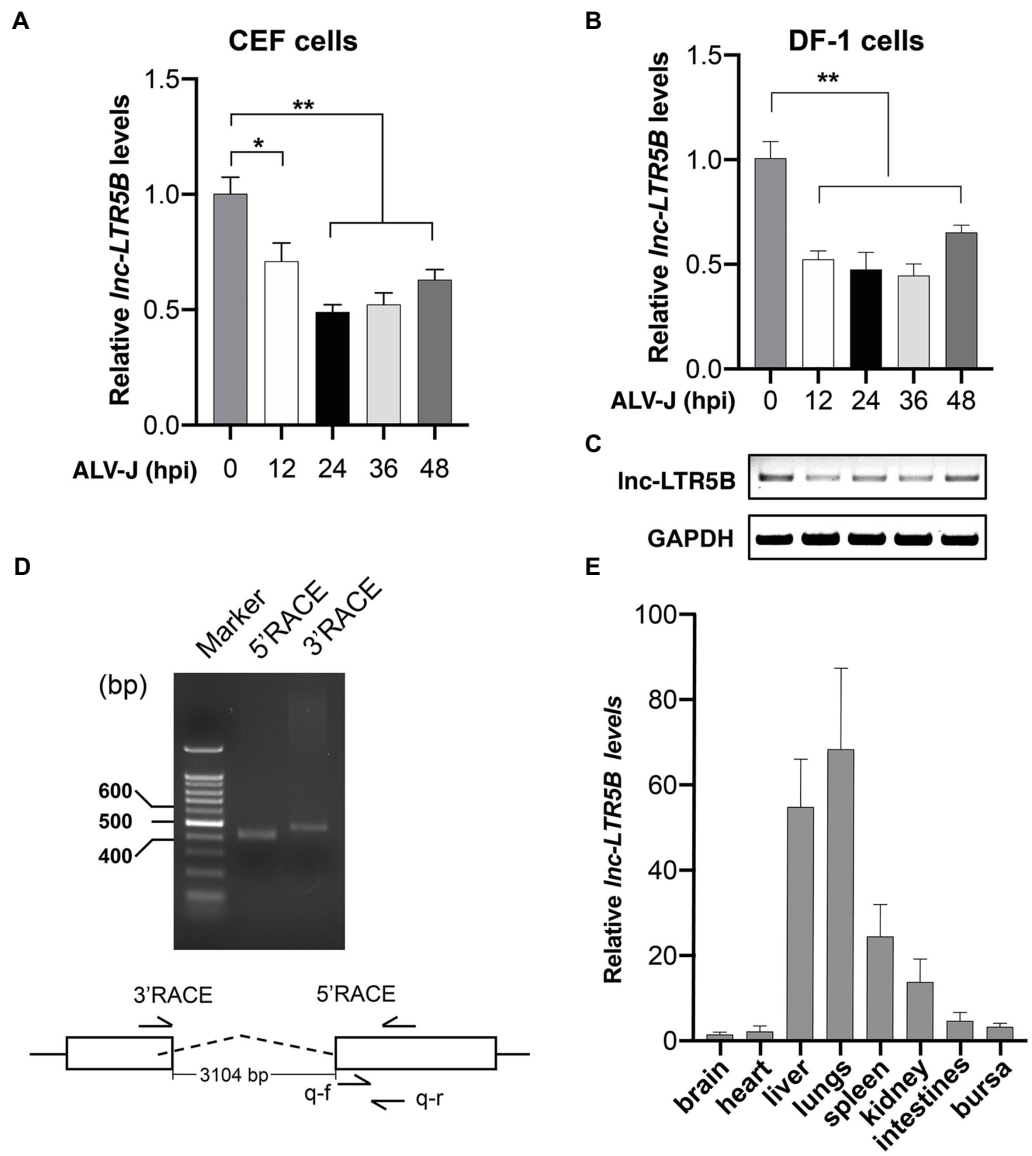
ALV-J infection downregulates lnc-LTR5B expression. **(A,B)** CEF cells **(A)** and DF-1 **(B)** cells were infected with ALV-J (Multiplicity of infection, MOI=0.5) for the indicated time intervals. The relative levels of lnc-LTR5B were measured by qRT-PCR. **(C)** RT-PCR analysis of the expression of lnc-LTR5B in DF-1 cells treated as described in **(B)**. **(D)** Agarose gel analysis of 5' and 3' RACE PCR products for lnc-LTR5B. DNA ladders (400bp, 500bp, and 600bp) are shown (left lane). **(E)** qRT-PCR analysis of the expression of lnc-LTR5B in multiple tissues in chicken. GAPDH mRNA served as an internal control to normalize the level of lnc-LTR5B. Data are presented as the mean±SD, *n*=3; ^*^p<0.05, ^**^p<0.01 (one-way ANOVA).

We first examined lnc-LTR5B expression during ALV-J infection using reverse transcription quantitative PCR (qRT-PCR) and confirmed its downregulation upon ALV-J infection of primary CEFs (Figure 1A). Subsequently, we examined the expression of lnc-LTR5B in DF-1 cells infected with ALV-J. qRT-PCR and semi-quantitative RT-PCR showed that lnc-LTR5B was downregulated during ALV-J infection (Figures 1B,C). To identify the full-length sequence of lnc-LTR5B, we performed 5' and 3' rapid amplification of cDNA ends (RACE; Figure 1D) and the sequencing result show that the full-length transcript was 590 nucleotides (Supplementary Table S2). Subsequently, its protein-coding potential was assessed using the Coding Potential Calculator (CPC) program. The data showed that the CPC score for lnc-LTR5B was −0.980, which was similar to that of the validated lncRNA XIST (−0.945), suggesting that lnc-LTR5B lacks coding potential (Supplementary Figure S1). We further measured the relative abundance of lnc-LTR5B in several chicken tissues. Our results suggest that lnc-LTR5B is detectable in the brain, heart, liver, lung, spleen, kidney, intestines, and bursa. In addition, lnc-LTR5B was highly expressed in the lung and liver tissues, with a relatively low expression level in the brain tissues (Figure 1E).

### Characterization of lnc-LTR5B

**Figure 2:**
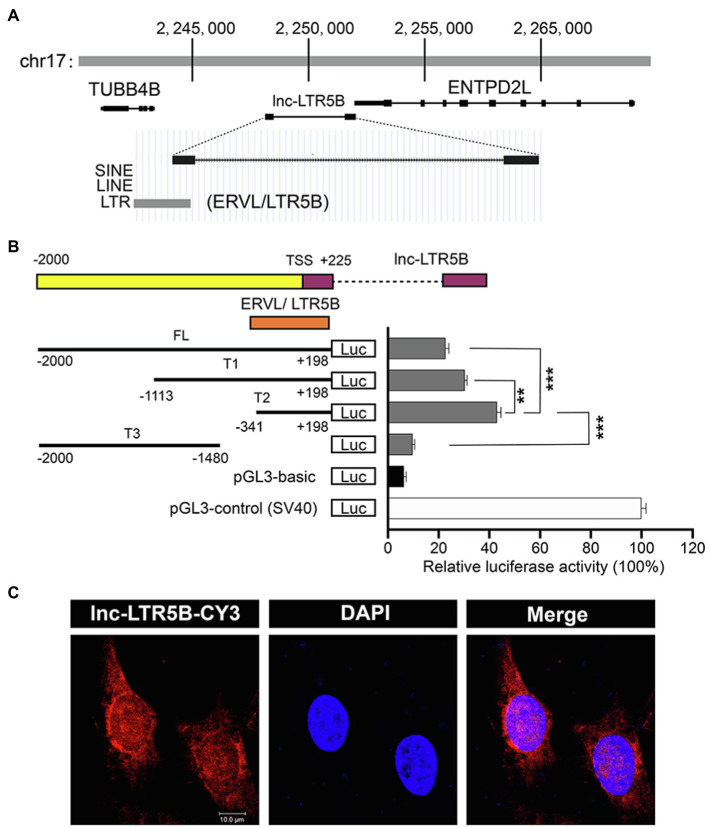
Characterization of the lnc-LTR5B. **(A)** Schematic map of lnc-LTR5B. lnc-LTR5B is located on chromosome 17 and is flanked by the coding genes TUBB4B and ENTPD2L. **(B)** Dual-luciferase assays of lnc-LTR5B promoter activity in HEK293T cells transfected with the indicated plasmids. Schematic illustration of truncation constructs of lnc-LTR5B promoter region (−2000 to +198 relative to TSS). Data are presented as the mean±SD, *n*=3; ^**^p<0.01, ^***^p<0.001 (two-tailed unpaired Student’s t test). **(C)** Confocal RNA FISH images showing the distribution of lnc-LTR5B in DF-1 cells. The lnc-LTR5B probes labeled with CY3 (red), and nuclei are stained with 4', 6-diamidino-2-phenylindole (DAPI; blue), Scale bars, 10μm.

BLAST analysis of the chicken genome from the UCSC Genome Browser^1^ database showed that lnc-LTR5B is located on chicken chromosome 17 and has two exons and is flanked by the upstream and downstream protein-coding genes TUBB4B and ENTPD2L (Figure 2A). Interestingly, the first exon of lnc-LTR5B was shown to be derived from an LTR5B element, which belongs to the ERV-L LTR family. Next, a dual luciferase assay was performed to verify whether lnc-LTR5B is an LTR-activated lncRNA. We inserted the full-length (from −2000 to +198) lnc-LTR5B promoter and truncated promoter fragments into a luciferase reporter vector. The luciferase activity of the T2 promoter region (from −341 to +198) was higher than that of the other promoter fragments (Figure 2B). The results indicate that lnc-LTR5B is an ERV LTR-derived lncRNA and is a member of the ERV-L lncRNA family. Next, we analyzed the subcellular localization of lnc-LTR5B by performing RNA fluorescence *in situ* hybridization (FISH). The results indicated that lnc-LTR5B is localized in both cytoplasm and nucleus, with distinct perinuclear aggregation (Figure 2C). Altogether, we identified that lnc-LTR5B is a novel LTR-derived lncRNA and its expression is suppressed in chicken cells infected with ALV-J.

### lnc-LTR5B Inhibits ALV-J Replication

**Figure 3:**
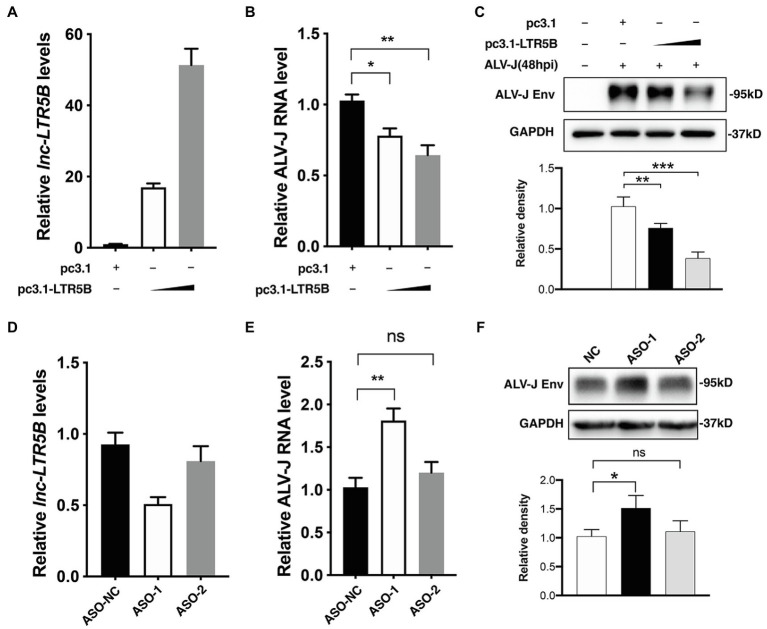
lnc-LTR5B restricts ALV-J replication in DF-1 cells. **(A-C)** Overexpression of lnc-LTR5B suppressed ALV-J production. DF-1 cells were transfected with increasing amounts of pcDNA3.1-lnc-LTR5B or control pcDNA3.1 for 12h, followed by infection with ALV-J (MOI=0.5). Forty-eight hours post-infection, cells were harvested, and the level of lnc-LTR5B **(A)** and viral RNA **(B)** was measured by qRT-PCR. **(C)** ALV-J Env was measured by western blotting (top), and the density of each band was analyzed using Image J software. (bottom). **(D-F)** Knockdown of lnc-LTR5B in DF-1 cells promoted ALV-J production. DF-1 cells were transfected with either negative control ASO (ASO-NC) or ASO against lnc-LTR5B (50nM) for 36h. **(D)** The levels of lnc-LTR5B were quantified by qRT-PCR to examine the silencing efficiency. The cells transfected with ASO were then infected with ALV-J (MOI=0.5) for 36h. **(E)** The viral RNA was determined by qRT-PCR. **(F)** ALV-J Env was measured by western blotting (top), and the density of each band was quantified by ImageJ software. (bottom). Expression data were normalized to those of the negative vector or ASO-NC group. Data are presented as the mean±SD, *n*=3; ^*^p<0.05, ^**^p<0.01, ^***^p<0.001, ns, not significant (one-way ANOVA).

To investigate the role of lnc-LTR5B in ALV-J replication, we transfected DF-1 cells with either negative control vector pcDNA3.1 or the lnc-LTR5B expression vector and infected them with ALV-J at 12h post-transfection. At 48 hpi, the cells were harvested for immunoblotting and qRT-PCR analysis. With increasing levels of lnc-LTR5B (Figure 3A), ALV-J RNA levels and Env protein expression decreased in a dose-dependent manner (Figures 3B,C). Next, the impact of lnc-LTR5B silencing on viral replication was assessed. Two different sets (ASO-1 and ASO-2) of antisense oligonucleotides (ASO) targeting lnc-LTR5B or ASO-NC as a control were used. The results of qRT-PCR showed that DF-1 cells transfected with ASO-1 achieved the highest silencing efficiency (Figure 3D). Consistent with this, the levels of ALV-J RNA and protein Env expression were both significantly increased in the DF-1 cells transfected with ASO-1 compared to those in the ASO-2 and ASO-NC groups at 36hpi (Figures 3E,F). In addition, our data showed that overexpression of lnc-LTR5B did not have a significant effect on the activation of IFN signaling and its downstream gene, MX1 (Supplementary Figure S2A). Furthermore, the expression of lnc-LTR5B was not affected by poly(I:C)-induced IFN signaling (Supplementary Figure S2B). Together, these data suggest that lnc-LTR5B-mediated inhibition of ALV-J replication might not involve the activation of the innate immune response.

### Identification of BiP as a Binding Protein of lnc-LTR5B

**Figure 4:**
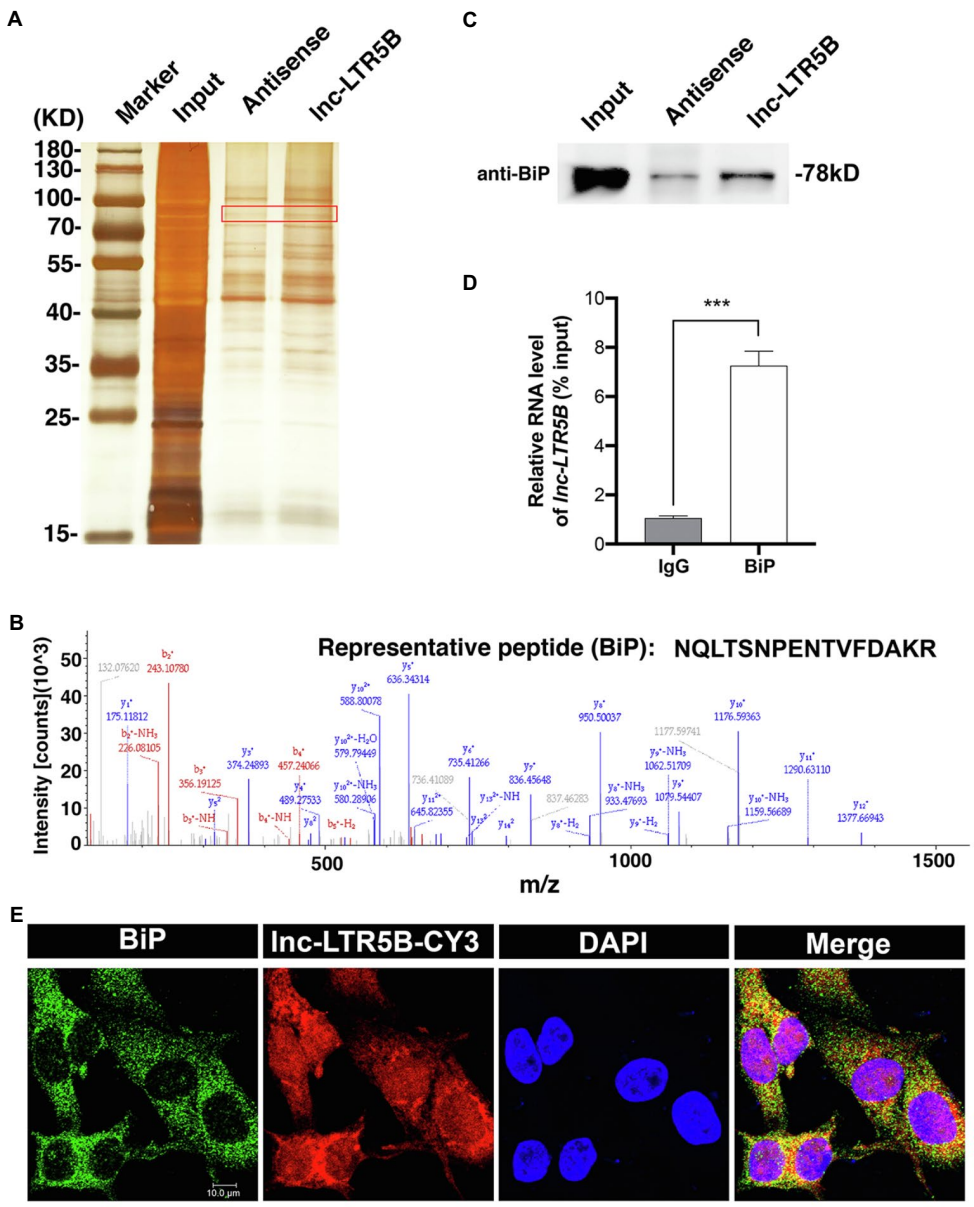
Identification of BiP as a binding protein of lnc-LTR5B. **(A)** Silver staining of biotinylated lnc-LTR5B-associated proteins. The lnc-LTR5B-specific bands (highlighted bands) were excised and analyzed by mass spectrometry. **(B)** Mass spectrometry of highlighted band digests identifies the fragment ions from the representative peptide of BiP protein. **(C)** Western blot analysis of BiP from RNA pulldown assay using biotinylated lnc-LTR5B or antisense RNA. **(D)** RNA immunoprecipitation (RIP)-qPCR analysis of lnc-LTR5B immunoprecipitated by BiP antibody from DF-1 cells. Data are presented as the mean±SD, *n*=3; ^***^p<0.001 (two-tailed unpaired Student’s t test). **(E)** RNA FISH detecting endogenous lnc-LTR5B (red) combined with immunofluorescence staining of BiP (green) in DF-1 cells. DAPI staining is shown in blue. Scale bars, 10μm.

Because lncRNAs can exert their functions through RNA-protein interactions to modulate target genes, we attempted to identify the proteins that bind lnc-LTR5B to explore the mechanism by which lnc-LTR5B restricts ALV-J replication. Therefore, we used RNA pull-down assays followed by silver staining and mass spectrometry (MS) to identify the protein partners of lnc-LTR5B (Figure 4A). MS analysis of the differentially displayed bands associated with lnc-LTR5B revealed that BiP is a potential protein that binds to lnc-LTR5B (Figure 4B). Subsequently, significant pulldown of BiP by the biotin-labeled sense lnc-LTR5B compared with the antisense sequence was confirmed by western blotting (Figure 4C). Next, the interactions between lnc-LTR5B and BiP were verified using RNA immunoprecipitation (RIP) assays (Figure 4D). Moreover, RNA FISH and immunofluorescence assays demonstrated the colocalization of lnc-LTR5B and BiP in the cytoplasm of DF-1 cells (Figure 4E).

### ALV-J Infection Induces the Translocation of BiP to the DF-1 Cell Surface

**Figure 5:**
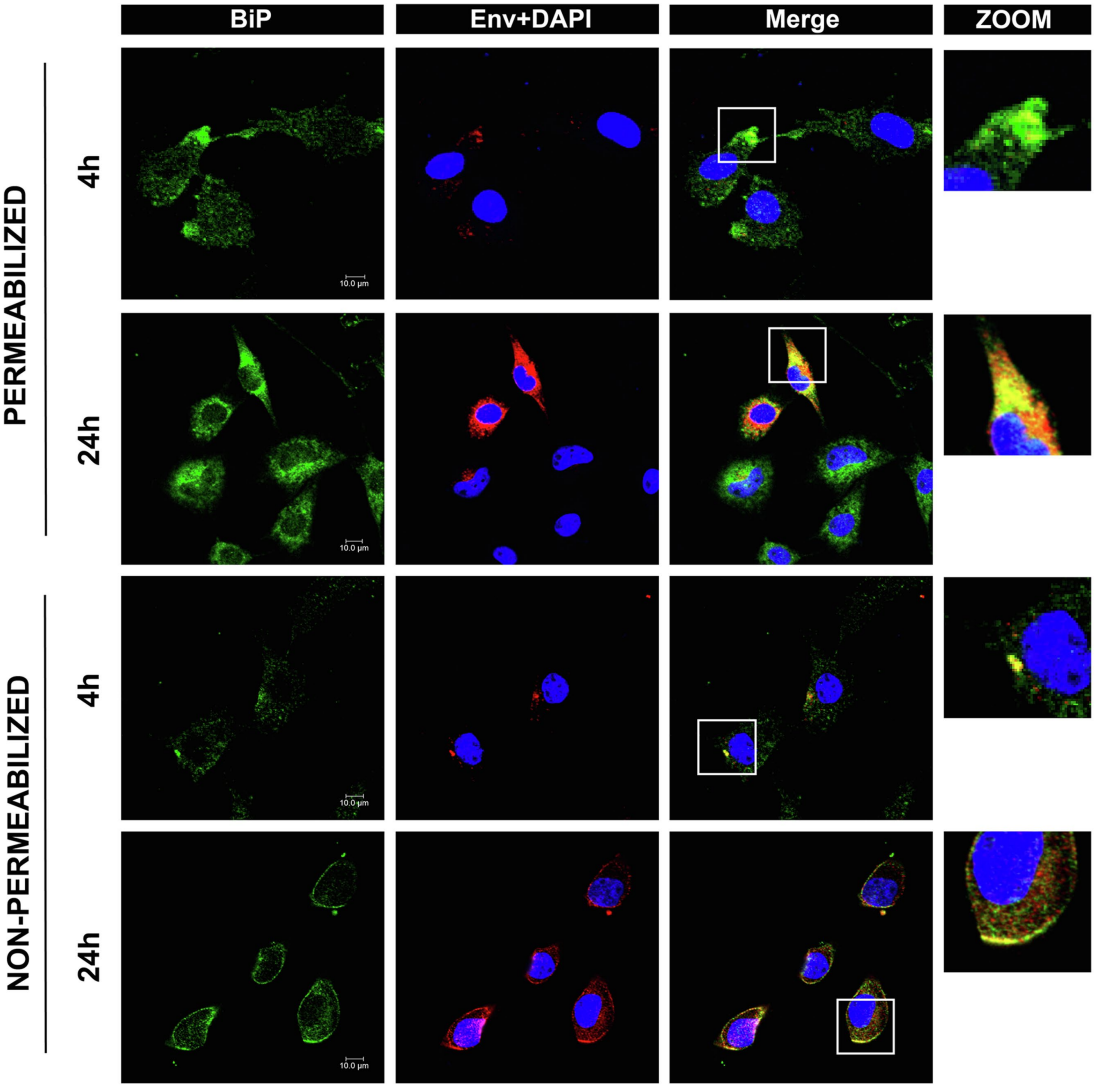
Confocal immunofluorescence shows BiP interactions with ALV-J Env protein. DF-1 cells grown on glass coverslips were infected with ALV-J at an MOI of 5 for the indicated time. The cells were fixed, permeabilized, or non-permeabilized, followed by immunofluorescence labeling of BiP (green) and ALV-J Env (red). DAPI (blue) indicates nuclear staining. Yellow indicates colocalization (scale bars, 10μm).

Because the lnc-LTR5B interacting protein BiP was reported as an entry receptor for ALV-J, it must be located on the plasma membrane of DF-1 cells. However, the expression levels of cell surface BiP are generally considered to be low under normal physiological conditions. The translocation of BiP to the cell surface can be observed under conditions of cellular stress, including viral infection (Gonzalez-Gronow et al., 2021). To examine whether ALV-J infection induces the translocation of BiP to the DF-1 cell surface, we performed immunocytochemical staining to analyze the distribution patterns of BiP in both permeabilized and non-permeabilized cells (Figure 5). We observed colocalization of intracellular BiP and ALV-J Env, implying that BiP plays an important role in ALV-J infection through its direct interaction with the ALV-J protein. In addition, we found that BiP colocalized on the cell surface with ALV-J Env, and the colocalization was stronger at 24hpi compared to that at 4 hpi, suggesting that ALV-J infection induces BiP translocation to the cell surface during ALV-J infection.

### lnc-LTR5B Inhibits ALV-J by Decreasing Surface Expression of Bip and Promoting Apoptosis of Infected Cells

**Figure 6:**
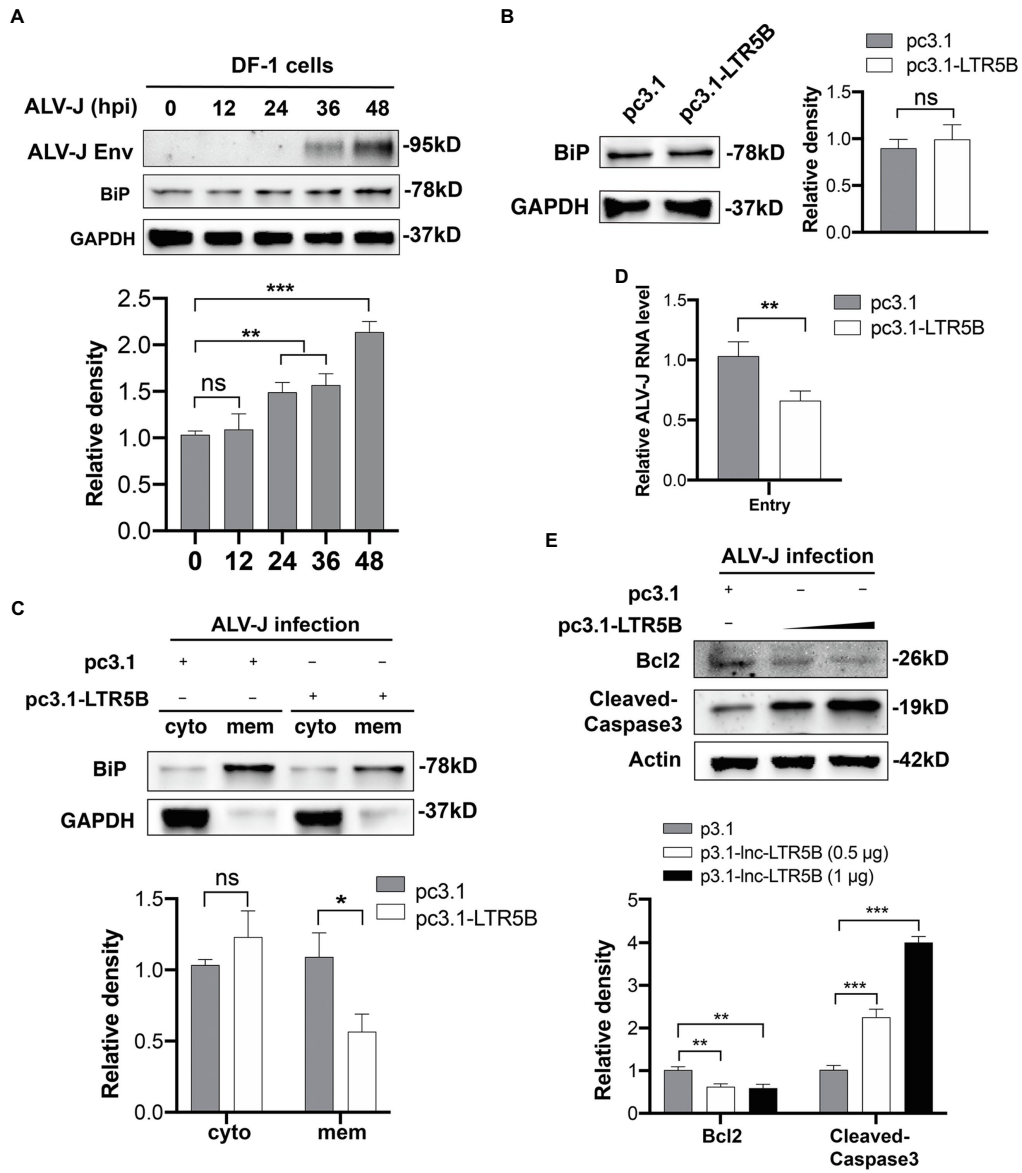
lnc-LTR5B inhibits ALV-J by decreasing surface expression of BiP and promoting apoptosis of infected cells. **(A)** Western blot analysis of BiP expression in DF-1 cells infected with ALV-J for the indicated time. **(B)** Western blot analysis of BiP expression in DF-1 cells transfected with either pcDNA3.1-lnc-LTR5B or control vector. **(C)** Western blot analysis of cell surface distribution of BiP in DF-1 cells transfected with either pcDNA3.1-lnc-LTR5B or control for 24h, followed by infection with ALV-J at MOI 5 for another 24h. GAPDH served as markers for the plasma membrane and cytosol fractions. **(D)** DF-1 cells were transfected with pcDNA3.1-lnc-LTR5B or control vector for 24h, followed by infection with ALV-J at MOI 20 on ice. At 1h post-infection, the cells were cultured at 37°C for another 1h. Then, the endocytosed-virus levels were determined by qRT-PCR for ALV-J genomic RNA. **(E)** Western blot analysis of Bcl2 and cleaved-caspase3 in DF-1 cells transfected with increasing amounts of pcDNA3.1-lnc-LTR5B or control pcDNA3.1, followed by infection with ALV-J at MOI 0.5 for 48h. The relative intensities of the different protein bands were analyzed and quantified by ImageJ software (**A,C,E**, lower panel; **B**, right panel). Data are presented as mean±SD, *n*=3; ^*^p<0.05, ^**^p<0.01, ^***^p<0.001, ns, not significant. Statistical analysis was carried out by one-way ANOVA **(A)** or two-tailed unpaired Student’s *t*-test **(B,C,E)**.

Next, we sought to find an association between lnc-LTR5B and BiP. Therefore, we quantified the expression of the BiP protein in DF-1 cells during ALV-J infection. The results of western blotting revealed that the expression of BiP was unchanged during the ALV-J infection within 12h, followed by a slight increase at 24hpi (Figure 6A). However, the expression of lnc-LTR5B began to decrease at 12hpi, the time point at which we first detected the expression of lnc-LTR5B (Figure 1B). Meanwhile, the overexpression of lnc-LTR5B did not affect the protein expression of BiP (Figure 6B). These data suggest that lnc-LTR5B is not directly involved in the regulation of BiP expression.

Because lnc-LTR5B binds BiP in the cytoplasm, we deduced that lnc-LTR5B can affect the BiP action that may be involved in BiP translocation to the cell surface. Western blot analysis of membrane fractions was performed to assess the effect of lnc-LTR5B overexpression on the surface expression of BiP upon ALV-J infection. As presented in Figure 6C, the expression of BiP on the membrane was attenuated in DF-1 cells overexpressing lnc-LTR5B compared to that in the corresponding control. Following this observation, we examined ALV-J binding and entry in lnc-LTR5B overexpressed DF-1 cells. As expected, we found that the overexpression of lnc-LTR5B markedly reduced the entry of ALV-J (Figure 6D). In addition, cell surface BiP has been reported to promote cell survival, which is considered an important target for cancer treatment (Araujo et al., 2018; Samanta et al., 2021). We speculate that the reduction of cell surface BIP caused by lnc-LTR5B leads to increased apoptosis. To test this, we assessed the effect of lnc-LTR5B overexpression on cell apoptosis signaling following ALV-J infection. The data showed that lnc-LTR5B overexpression inhibited the expression of the anti-apoptotic protein Bcl-2 and increased caspase-3 activation (Figure 6E), suggesting that lnc-LTR5B is involved in the activation of apoptotic signaling pathways in ALV-J-infected cells.

## Discussion

Many studies have highlighted the functional role of lncRNAs in several infectious diseases. Viruses can utilize lncRNAs to regulate the signaling pathways involved in innate immunity (Wang et al., 2020), cell metabolism (Khatun et al., 2020), and cell stress (Chattopadhyay et al., 2021), to survive and replicate in host cells. Like other viruses, it is conceivable that some lncRNAs for virus replication that are regulated by ALV-J infection exist, which has not been documented.

In the present study, lnc-LTR5B was discovered as a novel chicken lncRNA, which was markedly downregulated in ALV-J-infected cells. We demonstrated that overexpressed lnc-LTR5B exhibited an inhibitory effect on ALV-J replication *in vitro*. Notably, we identified that lnc-LTR5B is a long terminal repeat (LTR)-derived lncRNA, whose promoter and first exon are mostly present within an LTR5B element of ERV-L LTR family (Figures 2A,B). Research has shown that solitary LTRs are frequently “exonized” into novel lncRNAs (Kapusta et al., 2013). For example, lncRNA TROJAN was shown to promote breast cancer progression, and its sequence highly overlaps that of a long terminal repeat, LTR70 (Jin et al., 2019). The lncRNA PRLH1 is derived from the human LTR element LTR12C and regulates hepatocellular carcinoma progression (Deng et al., 2019). Solitary LTRs probably contain regulatory elements that are likely promoter, enhancer, and transcriptional binding sites, which act in cis to activate the transcription of downstream genes (Rebollo et al., 2012). Therefore, we speculated that transcriptional factors might participate in the regulation of lnc-LTR5B expression. This will be interesting to explore in future studies.

lncRNAs exert their regulatory functions through distinct mechanisms that are closely related to their cellular localization. For example, nuclear lncRNAs tend to control the chromosome architecture or the epigenetic state of genes, whereas cytoplasmic lncRNAs may function as competing endogenous RNAs by serving as sponges that bind miRNAs or proteins (Kim et al., 2016). To dissect the antiviral action of lnc-LTR5B against ALV-J, we first examined its cellular distribution and found that it was mainly expressed in the cytoplasm, suggesting that lnc-LTR5B may function at the post-transcriptional level. We then screened for proteins that may interact with lnc-LTR5B. The RNA pulldown and RNA immunoprecipitation (RIP) assay showed that lnc-LTR5B directly binds to BiP (Figures 4B,D). BiP, also known as GRP78, is encoded by the HSPA5 gene and belongs to the heat shock protein 70 (HSP70) family. As a typical chaperone in the endoplasmic reticulum (ER), BiP plays an essential role in controlling the unfolded protein response, which aims to restore ER homeostasis and promote cell survival (Wang et al., 2017).

Under ER stress conditions, BiP can be expressed at the cell surface, acting as a receptor for several signaling pathways, including anti-apoptotic and proliferative signals (Ni et al., 2011). Accumulating evidence has indicated that the translocation of BiP to the cell surface is associated with several pathological conditions, such as autoimmune diseases, cancers, and viral infections (Ni et al., 2011; Chu et al., 2018; Lenin et al., 2019). Viruses, such as SARS-CoV-2 (Carlos et al., 2021), were able to utilize cell surface BiP as an attachment factor mediating virus entry and pathogenesis. BiP has recently been described as a receptor for ALV-J entry into cells (Wang et al., 2016). Our data indicate that ALV-J causes BiP translocation to the cell surface, where the ALV-J envelope glycoprotein recognizes BiP, and mediates virus entry into host cells. Importantly, some BiP actions depend on its interaction with other proteins. For example, BiP surface translocation is required for different cotransporters, such as DNAJC3 (Vig et al., 2019), Par-4 (Cohen et al., 2013), and MTJ-1 (Misra et al., 2005), in a context-dependent manner. Recent studies have indicated that lncRNAs can directly interact with the molecular chaperone HSP90, regulating signaling or protein stability by altering chaperone function (Guo et al., 2018; Cui et al., 2020). Our data indicated that lnc-LTR5B binds to BiP in the cytoplasm. We investigated the potential influence of this interaction on BiP translocation to the cell surface. As expected, our data show that overexpression of lnc-LTR5B decreased BiP expression on cell surface induced by ALV-J infection in DF-1 cells (Figure 6C). Accumulating evidence has indicated that cell surface BiP is also implicated in pro-survival signals; an antibody that blocked cell surface BiP was used as an anti-tumor therapeutic (Shu et al., 2020). Consistent with this, we found lnc-LTR5B overexpression inhibited the expression of the anti-apoptotic protein Bcl-2 and increased caspase-3 activation, suggesting the activation of apoptotic signaling pathways. In addition, the cytoplasmic colocalization of BiP and ALV-J Env was observed in this study, and the impact of lnc-LTR5B on this interaction requires further investigation.

**Figure 7:**
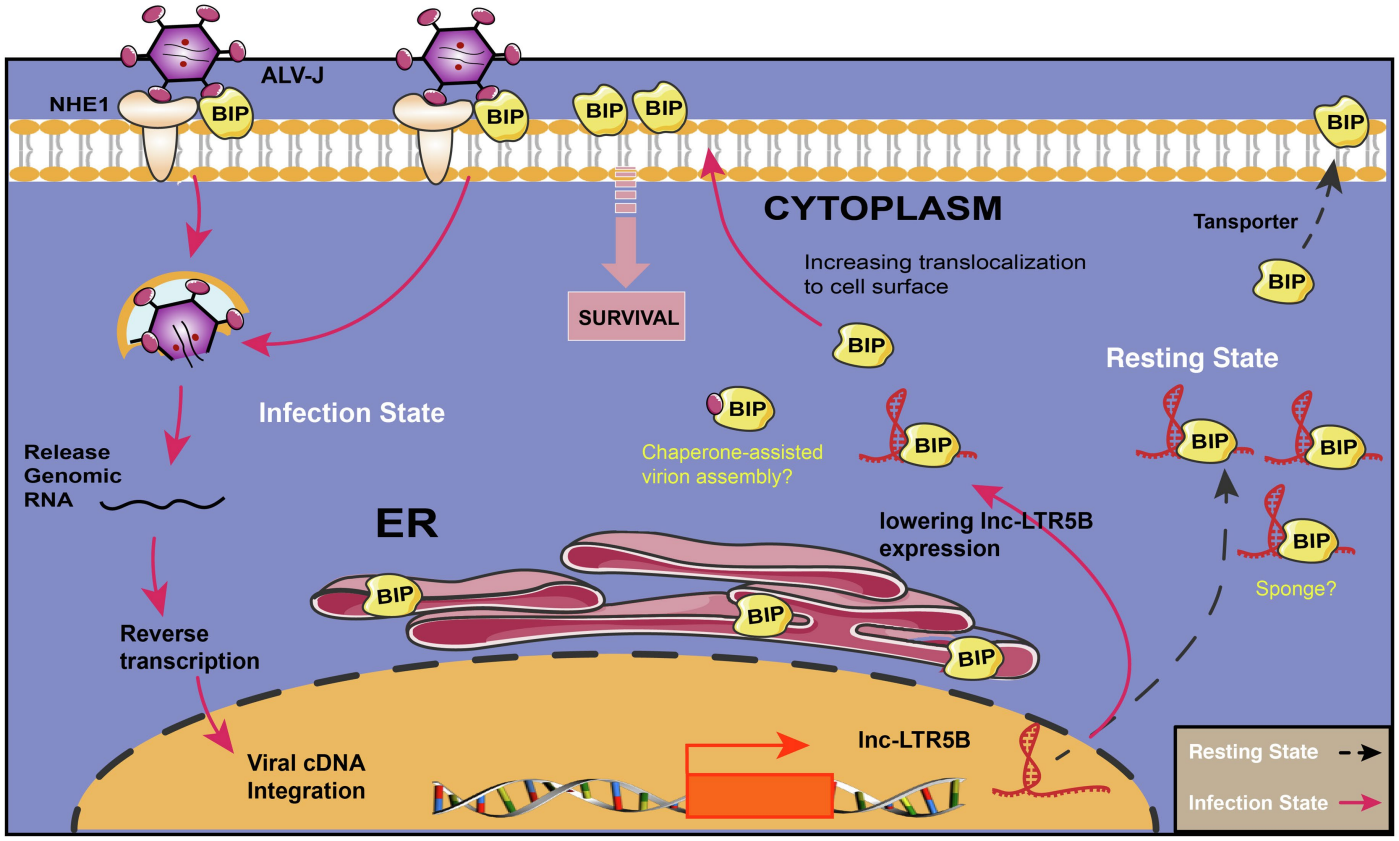
Schematic diagram showing the proposed mechanism for the effects of lnc-LTR5B on regulating ALV-J infection *via* BiP. In resetting cells, lnc-LTR5B localizes in the cytoplasm where it is abundant and binds free BiP to control cell homeostasis. Upon ALV-J infection, lnc-LTR5B expression is reduced, which favors the dissociation of BiP from its binding state, allowing its translocation to the cell surface, which is crucial for ALV-J entry as well as pro-survival signaling.

In summary, our study highlights the importance of lnc-LTR5B that modulates BiP cell surface translocation during ALV-J infection. In resetting cells, lnc-LTR5B expression is abundant and binds free BiP to control cell homeostasis; However, upon ALV-J infection, the expression of lnc-LTR5B is decreased, which releases BiP and allows its translocation to the cell surface; this is crucial for ALV-J entry and pro-survival signaling (Figure 7). We posit that the surface translocation of BiP is a critical process for cell survival in response to ALV-J infection (mild ER stress), while the presence of BiP at the cell surface might be exploited by ALV-J to complete its life cycle and propagate. Conversely, the overexpression of lnc-LTR5B sponges BiP, leading to cell apoptosis due to the interruption of the translocation process and prolonged ER stress. This study expands our knowledge of the LTR-derived lncRNAs in virus-host interactions and reveals novel cellular targets that have the potential to control ALV-J replication.

## Data Availability Statement

The original contributions presented in the study are included in the article/Supplementary Material, further inquiries can be directed to the corresponding authors.

## Ethics Statement

The animal study was reviewed and approved by the Committee on the Ethics of Animal Experiments of Yangzhou University, Jiangsu Province, China.

## Author Contributions

SC and HC designed the experiment. SC, RZ, TW, DW, SP, and BW performed experiments. SC, RZ, DW, XH, and ZP analyzed the data. SC and RZ wrote and edited the manuscript. HC supervised the research project. All authors reviewed and approved the final manuscript.

## Funding

This research was funded by the grant from the National Nature Science Foundation of China (32002271 and 91540117), the China Postdoctoral Science Foundation (2019M651986), and the Open Project Program of Jiangsu Key Laboratory of Zoonosis (R1911).

## Conflict of Interest

The authors declare that the research was conducted in the absence of any commercial or financial relationships that could be construed as a potential conflict of interest.

## Publisher’s Note

All claims expressed in this article are solely those of the authors and do not necessarily represent those of their affiliated organizations, or those of the publisher, the editors and the reviewers. Any product that may be evaluated in this article, or claim that may be made by its manufacturer, is not guaranteed or endorsed by the publisher.

## Footnotes

^1^ http://genome.ucsc.edu/
