# Supplementary material for: An Endogenous Retroviral LTR-Derived Long Noncoding RNA lnc-LTR5B Interacts With BiP to Modulate ALV-J Replication in Chicken Cells
Source: Front Microbiol. 2021 Nov 29;12:788317. doi: 10.3389/fmicb.2021.788317 (PMC8667585; doi:10.3389/fmicb.2021.788317)
Supplement: Supplementary file 1 [file Table_1.DOCX]

**Supplementary Figures**

**This file includes: Figures S1 and S2**


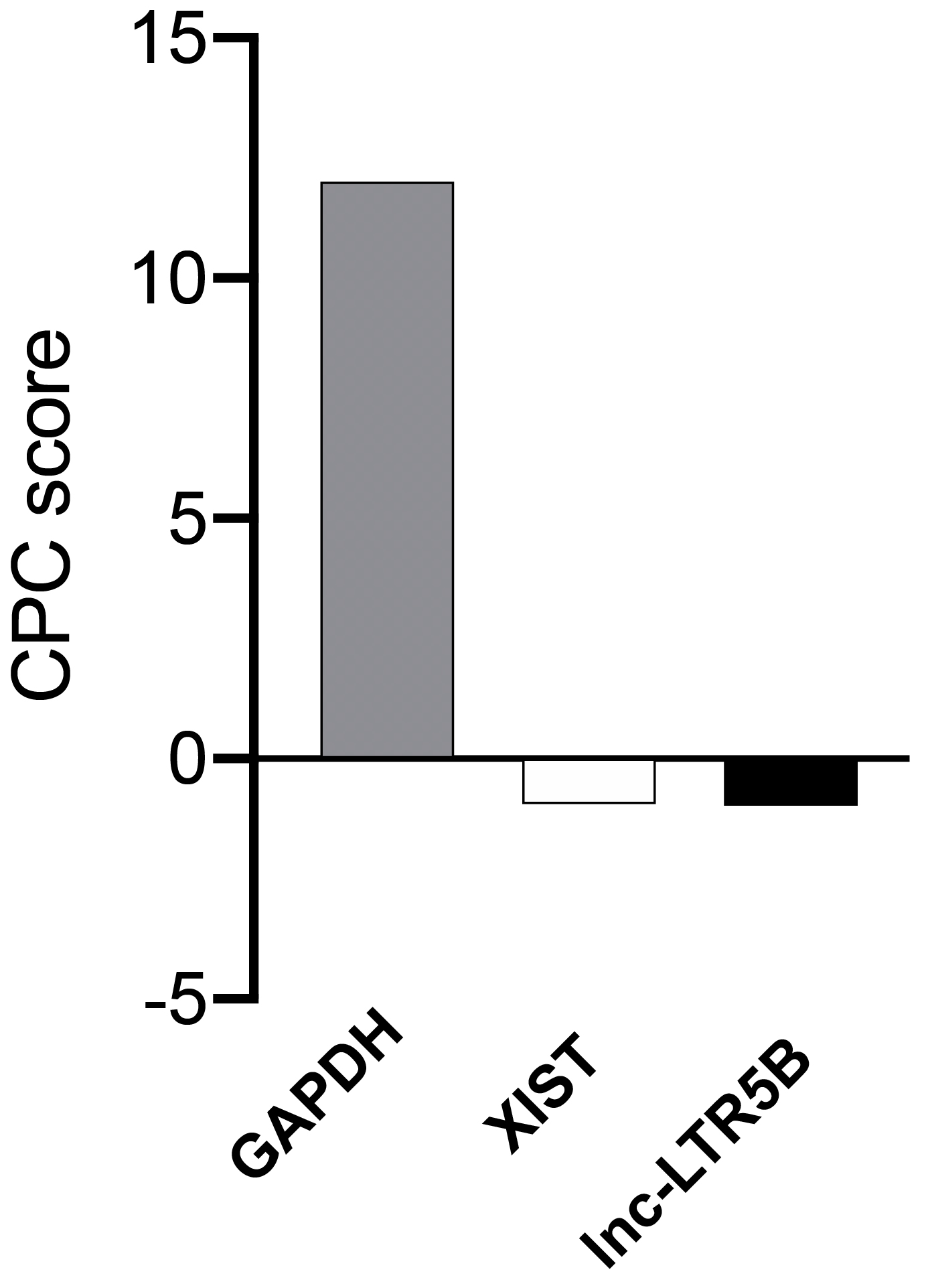


**Figures S1.** Analysis of protein-coding potential of Lnc-LTR5B. The sequence of GAPDH, XIST, and lnc-LTR5B were subjected to analyze the CPC score by the Coding Potential Calculator available at <http://cpc.cbi.pku.edu.cn/>. GAPDH and XIST were used as controls for protein-coding genes and noncoding RNAs, respectively. Gene with CPC score of less than zero was considered as non-coding genes.


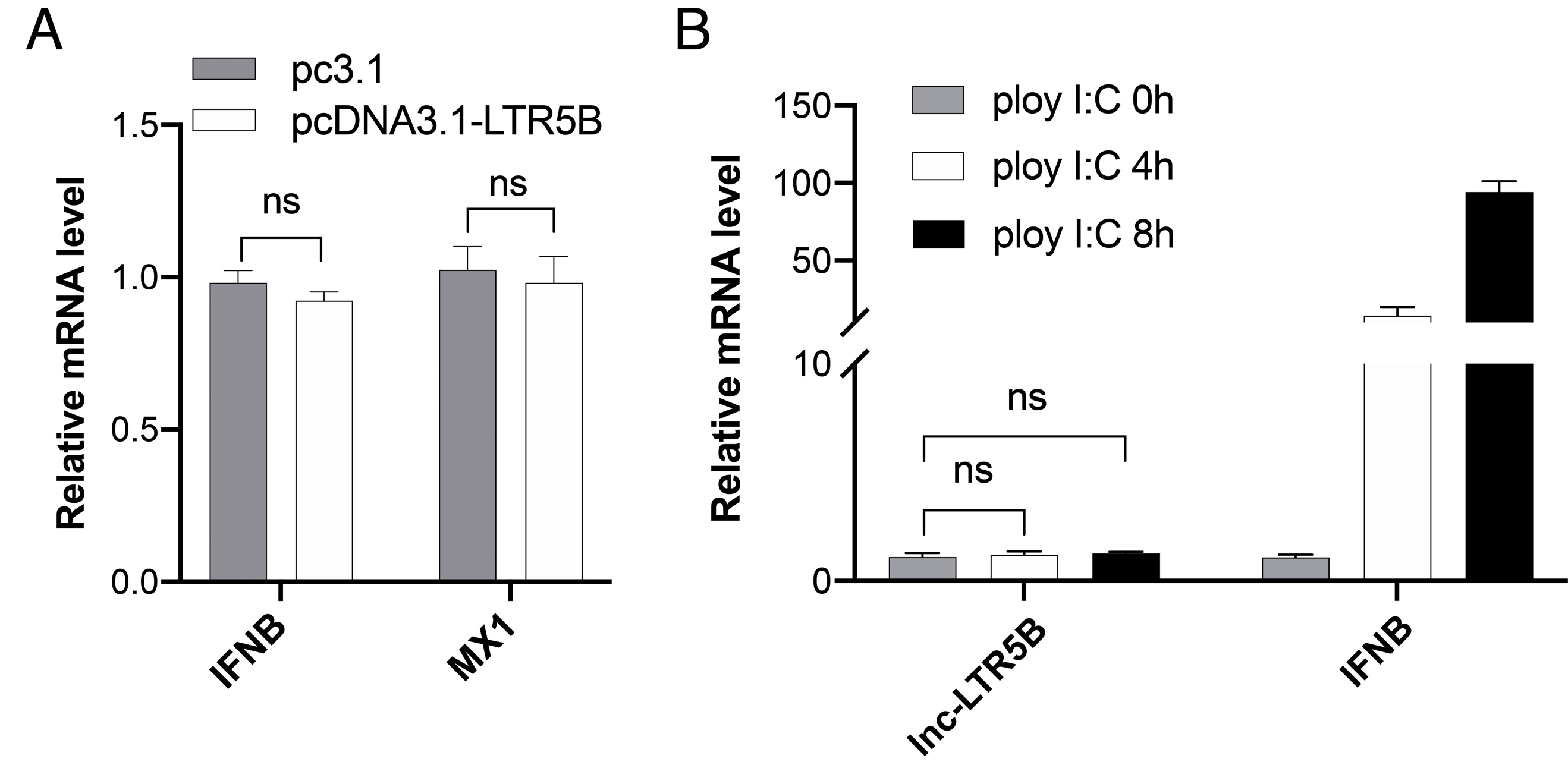


**Figures S2. (A)** DF-1 cells were transfected with either pcDNA3.1-lnc-LTR5B or control pcDNA3.1. After 36 h, cells were harvested, and the levels of IFNB and MX1 were measured by qRT-PCR. **(B)** qRT-PCR analyses of the expression of lnc-LTR5B and IFNB in DF-1 cells treated with poly (I:C) for indicated times. Data are presented as the mean ± SD, n=3; ns, no significant (two-tailed unpaired Student’s t test).
