# Supplementary material for: An Endogenous Retroviral LTR-Derived Long Noncoding RNA lnc-LTR5B Interacts With BiP to Modulate ALV-J Replication in Chicken Cells
Source: Front Microbiol. 2021 Nov 29;12:788317. doi: 10.3389/fmicb.2021.788317 (PMC8667585; doi:10.3389/fmicb.2021.788317)
Supplement: Supplementary file 2 [file Table_2.DOCX]

**Supplementary Tables**

**This file includes: Tables S1 and S2**

**Table S1. Primers used in this study**

| **qPCR Primer name** | **Nucleotide sequence (5'-3')** |
| --- | --- |
| lnc-LTR5B-F | AATCCCTCCTCTTCCTTCTT |
| lnc-LTR5B-R | GATAACTTGGCTGCTGGTA |
| GY3-Env-F | TTGGTTCGGTGTGCTATG |
| GY3- Env-R | GTCTCGTTGCTGGTGAAT |
| IFNB-F | GCCCACACACTCCAAAACACTG |
| IFNB-R | TTGATGCTGAGGTGAGCGTTG |
| MX1-F | CCGCAACACAGAAATACAG |
| MX1-R | TTATCTTGTGGCTGGTTCC |
| GAPDH-F | GAGAAACCAGCCAAGTATGA |
| GAPDH-R | CTGGTCCTCTGTGTATCCTA |

| **Primer for PCR** | **Nucleotide sequence (5'-3')** |
| --- | --- |
| RT-LTR5B-F | AAGCATAGCGTCATACCAG |
| RT-LTR5B-R | TGTTTCAGAGGCGAGGAT |
| 5’-RACE | CCAGTGGCAGGGAGGCAGAAAATGACCT |
| 3’-RACE | TGGTTGGGATCAAGCAGCAAGTCTATCC |
| P3.1-LTR5B-F | ACCCAAGCTGGCTAGCGTTTCTCTTGCTGGCTGCACAG |
| P3.1-LTR5B-R | GGCTGATCAGCGGGTTTTTTCTTTGAGTTGCAGGTTAGATATTTTTA |
| FL-F | GCTCTTACGCGTGCTAGTCCTGTGATTGAGTCTTTGG |
| FL-R | TACCGGAATGCCAAGCTCTGTTCCTCTTTCCTGGTTA |
| T1-F | GCTCTTACGCGTGCTAGGGAAGACATAGCAGTGAAGT |
| T2-F | GCTCTTACGCGTGCTAGTCTCTCACCGCCTTCAGT |
| T3-R | TACCGGAATGCCAAGCTGAAGGACTGGAATGACCA |
| T7-LTR5B-F | TAATACGACTCACTATAGGGCTCTTGCTGGCTGCACAG |
| T7-LTR5B-AS-F | TAATACGACTCACTATAGGGAGTTGCAGGTTAGATATTTTTA |
| ASO-1 targeting site | CTCATCAACTAAATACCAGC |
| ASO-2 targeting site | AACAACTCCTGAGTAAACAC |

**Table S2. The sequence of lnc-LTR5B expressed in this study**

| **CTCTTGCTGGCTGCACAGTGTGAGAAGCTGAAAAACTGCAACGTCTTTGGCTCTGTACAGTGCTGCTCAGCAAAAAACTAAAACATCAGTTTGTTCTTAGCAATGTTTTTCTCCTTAGGCAAAAGCATAGCGTCATACCAGGCACTATGAAGAAAATCGTCTCTGTTTCAGCTGTAACCAGGAAAGAGGAACAGCTGGTTGGGATCAAGCAGCAAGTCTATCCAGGCTTGTCTGAATCCCTCCTCTTCCTTCTTCAGATGGTTTGAGCAAAGAACACATCAAAGATGTTGACGTGTCT*CTCATCAACTAAATACCAGC*AGCCAAGTTATCTAAGGTCATTTTCTGCCTCCCTGCCACTGGGAAGAAAGTGCCTTCAGGGGTTCTCATTGTGTACCCCAGAAGTACACAGCAT*AACAACTCCTGAGTAAACAC*AGGCACAGTCAGCATCCCCCATCCTCGCCTCTGAAACAGTTTGTATTCACAATAAAACCCCTGCTTTGATCTCTTGCCAAGAATAACACAGACAGTCTATTCAAACATCTTTAATAACATGATATAATTAAAAATATCTAACCTGCAACTCAAAGAAA** |
| --- |

**The sequences in italics show the ASO targeting sites.**
